# System to Evaluate the Skill of Operating Hydraulic Excavators Using a Remote Controlled Excavator and Virtual Reality

**DOI:** 10.3389/frobt.2019.00142

**Published:** 2020-01-09

**Authors:** Ryota Sekizuka, Masaru Ito, Seiji Saiki, Yoichiro Yamazaki, Yuichi Kurita

**Affiliations:** ^1^Graduate School of Engineering, Hiroshima University, Hiroshima, Japan; ^2^Kobelco Construction Machinery Co., Ltd., Hiroshima, Japan

**Keywords:** skill evaluation, virtual reality, construction machine, remote controlled, dynamic time warping

## Abstract

We developed a system to evaluate the skill of operating a hydraulic excavator. The system employs a remotely controlled (RC) excavator and virtual reality (VR) technology. We remodeled the RC excavator so that it can be operated in the same manner as a real excavator and proceeded to measure the excavator's state. To evaluate the skill of operating this system, we calculated several indices from the data recorded during excavation work and compared the indices obtained for expert and non-expert operators. The results revealed that it is possible to distinguish whether an expert or non-expert is operating the RC excavator. We calculated the same indices from the data recorded during excavation with a real excavator and verified that there exists a high correlation between the indices of the RC excavator and those of the real excavator. Thus, we confirmed that the indices of the real excavator and those of the simulator exhibited similar trends. This suggests that it is possible to partly evaluate the operation characteristics of a real excavator by using an RC excavator with different dynamics compared with a real excavator.

## 1. Introduction

Hydraulic excavators are construction machines with working devices that have a high degree of freedom and various attachments. Thus, these excavators can be used for various types of work, such as excavation and demolition. The operators of hydraulic excavators should have advanced skills and experience. To operate an excavator as desired, the operator controls the levers in the cab; however, this type of operation is not very intuitive. In recent years, the number of workers in the construction industry has been decreasing, and the decrease in the number of young workers has posed a challenge. Hence, the early training of young hydraulic excavator operators is indispensable, but operation training with a real excavator may be difficult in terms of securing an appropriate location and ensuring safety. Simulators can be effective for training in situations wherein it is difficult to train using an actual machine in a real environment. Training can be performed safely by using a simulator and with lower operating cost than that incurred when using the actual machine. Various studies have dealt with training using simulators, and several training simulators have been developed for construction machinery (Kamezaki et al., 2008; Wang et al., 2011; Ni et al., 2013). Some studies have considered the dynamics of the hydraulic excavator in simulators (Vähä and Skibniewski, 1993; Patel and Prajapati, 2011; Vujic et al., 2017). There exists a challenge in the case of excavator simulators: It is difficult to reproduce the behavior of soil excavated in real time because of the calculation cost. We think that this problem can be solved by using a training system using a remote-controlled (RC) toy excavator.

Quantitative evaluation of operation skills is useful for training. Typically, the time spent on a work is used to evaluate the operating skills of hydraulic excavators, but this parameter is not sufficient for detailed skill evaluation. Research has been conducted on quantifying the operation skills of an excavator (Bernold et al., 2003; Sakaida et al., 2008; Koiwai et al., 2016). It is unknown, however, whether the skill evaluation method applied for a real excavator can be applied when using an RC toy excavator that cannot completely simulate the characteristics of actual excavators. Therefore, it is necessary to verify the indices that can quantitatively evaluate the operation skill regardless of whether the operation target is a real excavator or an RC toy excavator.

In this study, we developed a simulator that can measure each joint angle in real time by using an RC toy excavator with the same viewpoint and operating interface as a real excavator. We calculated the evaluation indices for lever operation and bucket movement using the proposed system and a real excavator and determined the indices of operation skills with high correlation between the RC toy excavator and real excavator. The preliminary experimental results were explained in IROS2019 (Sekizuka et al., 2019). The new contributions of this paper are a comparison of the behavior of our RC excavator and that of a real excavator, a subjective evaluation of the operability and the reality of the field of view of our RC excavator system, and verification of skill evaluation indices including a new index for lever operation.

## 2. Related Work

In order to increase the presence in a teleoperation system, a method of presenting images captured by a camera placed on the operation target to the user via a head mounted display (HMD) is adopted. For example, HMD-based mixed reality is used to enhance telepresence during the teleoperation of road vehicles and UAVs (Hosseini and Lienkamp, 2016; Sawarkar et al., 2016). It is also used for a teleoperated excavator (Ito et al., 2019). The same method is also used for training systems using alternative machines such as RC toys and smaller machines. An operation training system using an RC helicopter has been developed (Kunieda and Hoshino, 2009). This system provides a viewpoint from a cockpit in the helicopter by projecting images from a camera attached to the RC helicopter on an HMD and thus helps improve the reality of the operation. Override Ship Maneuvering Simulator is an actual training ship, and augmented reality (AR) technology has been developed (Takaseki et al., 2014). This system gives the sensation of being on a large ship by displaying a three-dimensional (3D) model of a large ship superimposed on the HMD image using an AR marker installed at the bow. In this study, this technique is used to reproduce the field of view as if riding an RC excavator.

## 3. Operation Training System Using an RC Excavator

**Figure 1:**
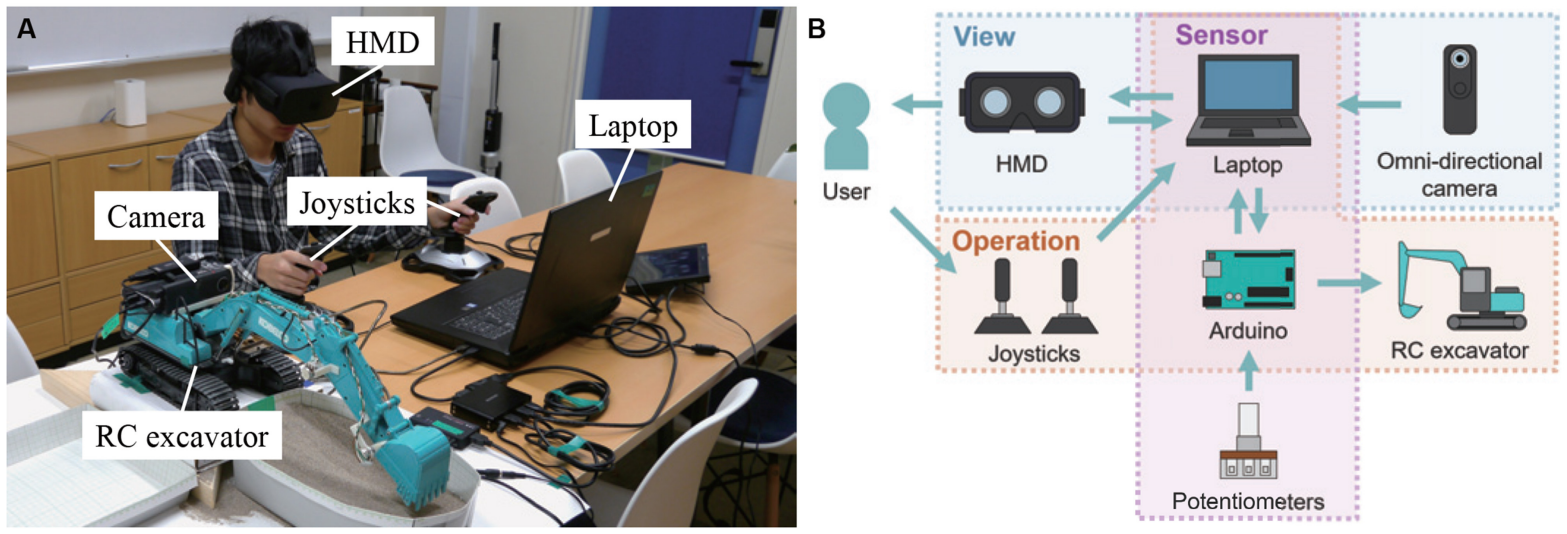
System using RC excavator and VR technology. **(A)** System appearance and **(B)** system configuration. Written informed consent was obtained from the individual for the publication of this image.

Figure 1 shows the overview and configuration of the developed system using an RC excavator and virtual reality (VR) technology. This system consists of an RC excavator, an omnidirectional camera (RICOH, RICOH THETA S), an HMD (Oculus VR, Oculus Rift CV 1), two joysticks (Logitech, Extreme 3D Pro), four variable resistors (Linkman, R1610N-QB1-B103), a microcomputer board (Arduino SRL, Arduino Uno; hereinafter, referred to as Arduino), and a laptop computer.

**Figure 2:**
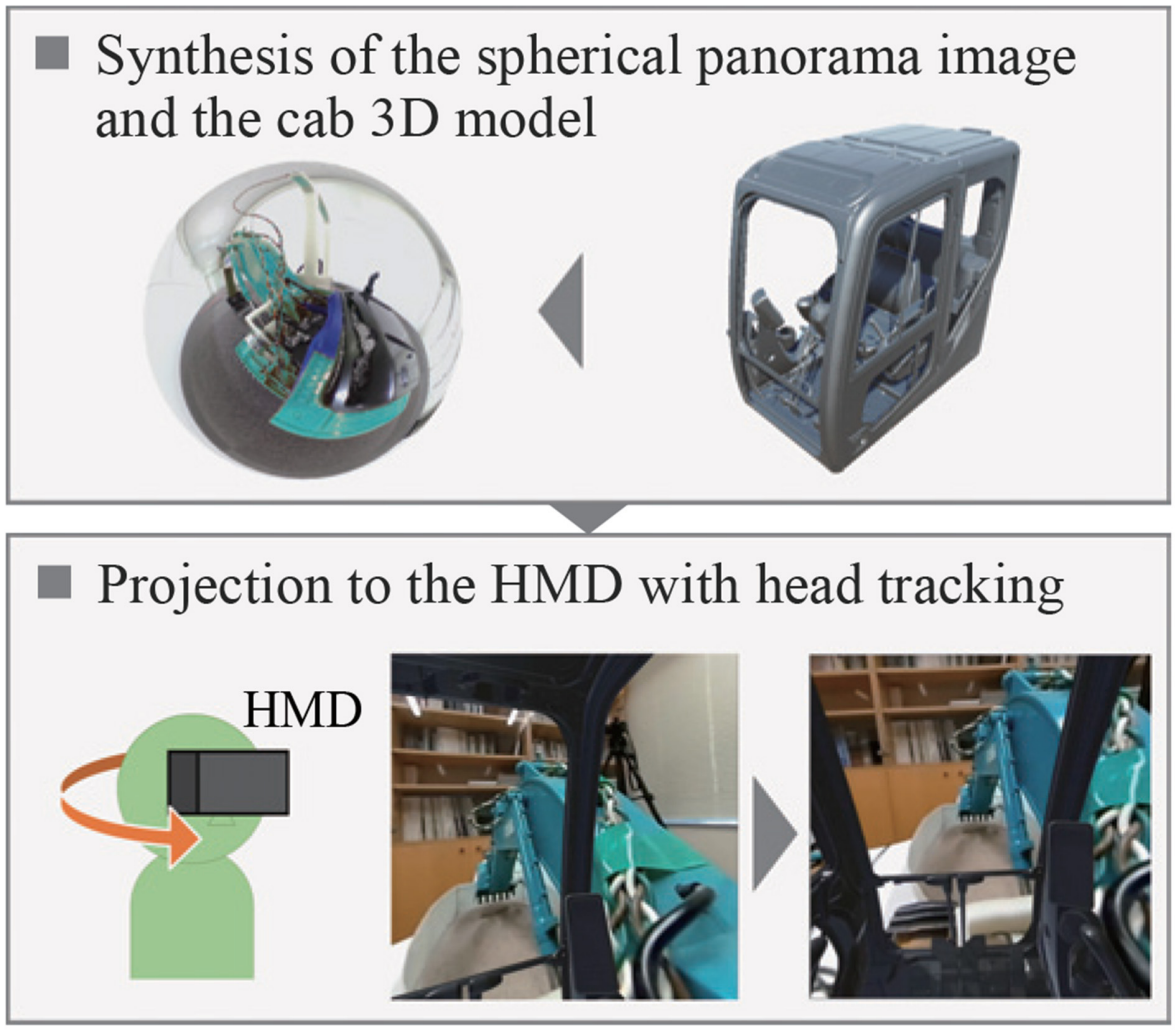
Reproduction of field of view from cab.

**Figure 3:**
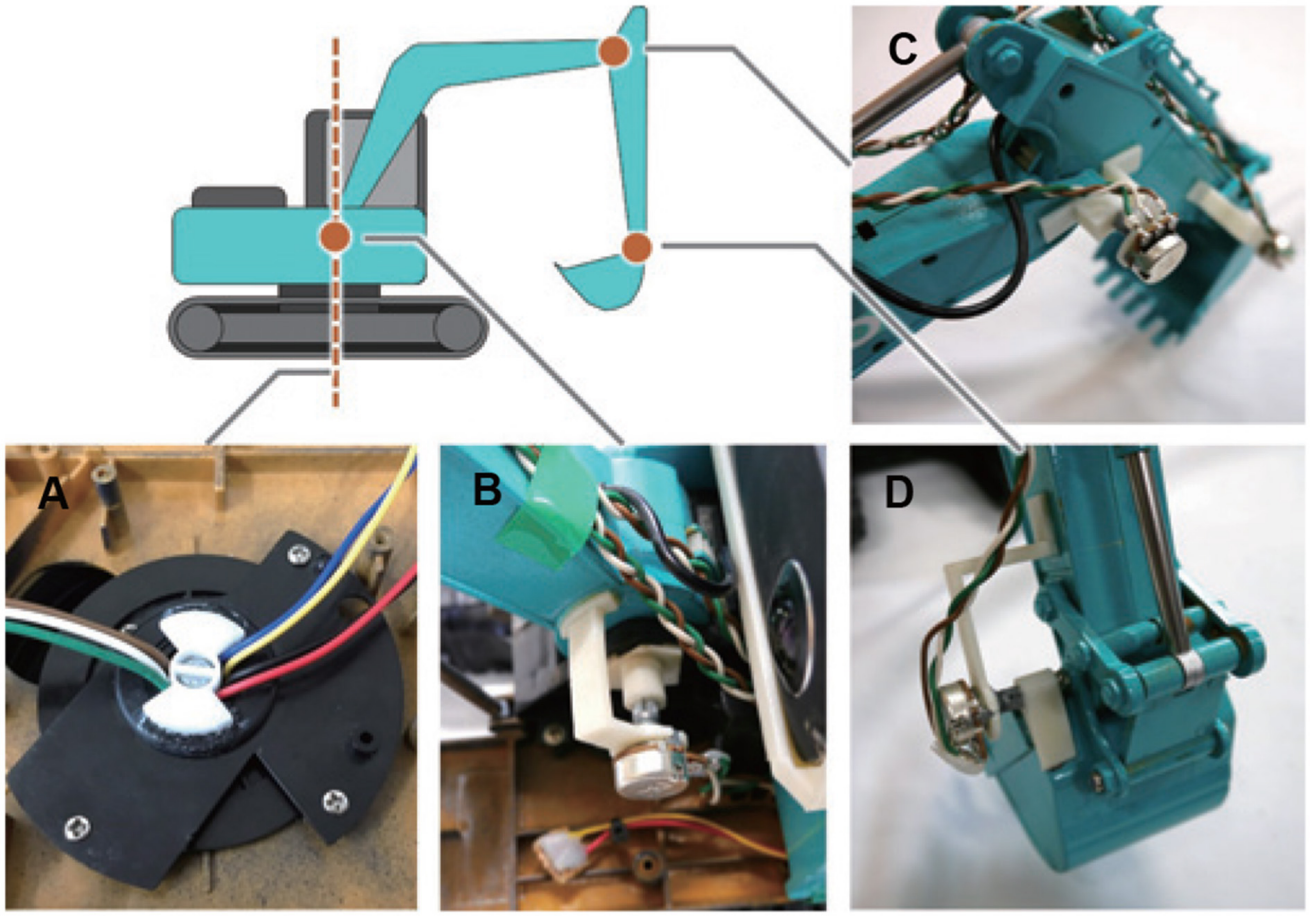
Mounting position of each potentiometer. **(A)** Swing joint, **(B)** boom joint, **(C)** arm joint, and **(D)** bucket joint.

The field of view from the cab of the excavator was reproduced in this system. Figure 2 shows the method of reproducing the field of view from the cab. The omnidirectional camera was mounted on the RC excavator. The obtained images were transmitted to the laptop over HDMI, and spherical panoramic images were generated. A three-dimensional (3D) model of the cab of a hydraulic excavator was arranged at the center of the spherical panoramic image. The field of view from the 3D model of the cab was projected onto the HMD and it corresponded to the orientation of the user through the head-tracking function of the HMD. In this way, the user can experience a field of view as if he were on the RC excavator. A series of processing of image presentation was performed using the game engine Unity. The delay time of the visual system was about 170 ms. We modified the RC excavator so that it could be operated with the two joysticks. Thus, it was possible to perform the same operation as that for the real excavator. Moreover, it was possible to measure each joint angle of the RC excavator during the operation. As shown in Figure 3, potentiometers were attached to the joints of the RC excavator. The control of the RC excavator and the measurement of the joint angles were realized by using the laptop and Arduino.

**Figure 4:**
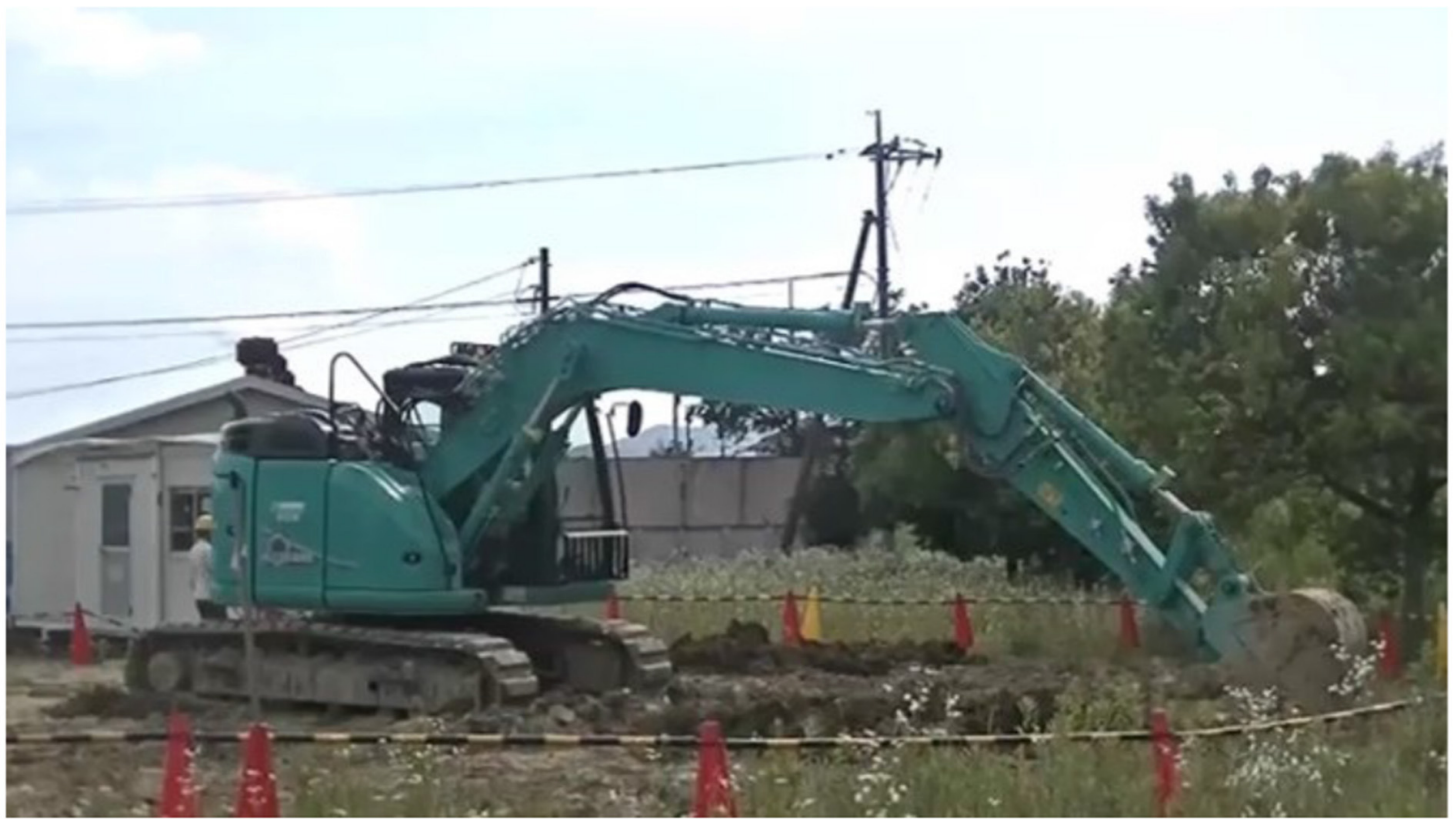
Real excavator (SK135SR-5, Kobelco construction machinery).

**Figure 5:**
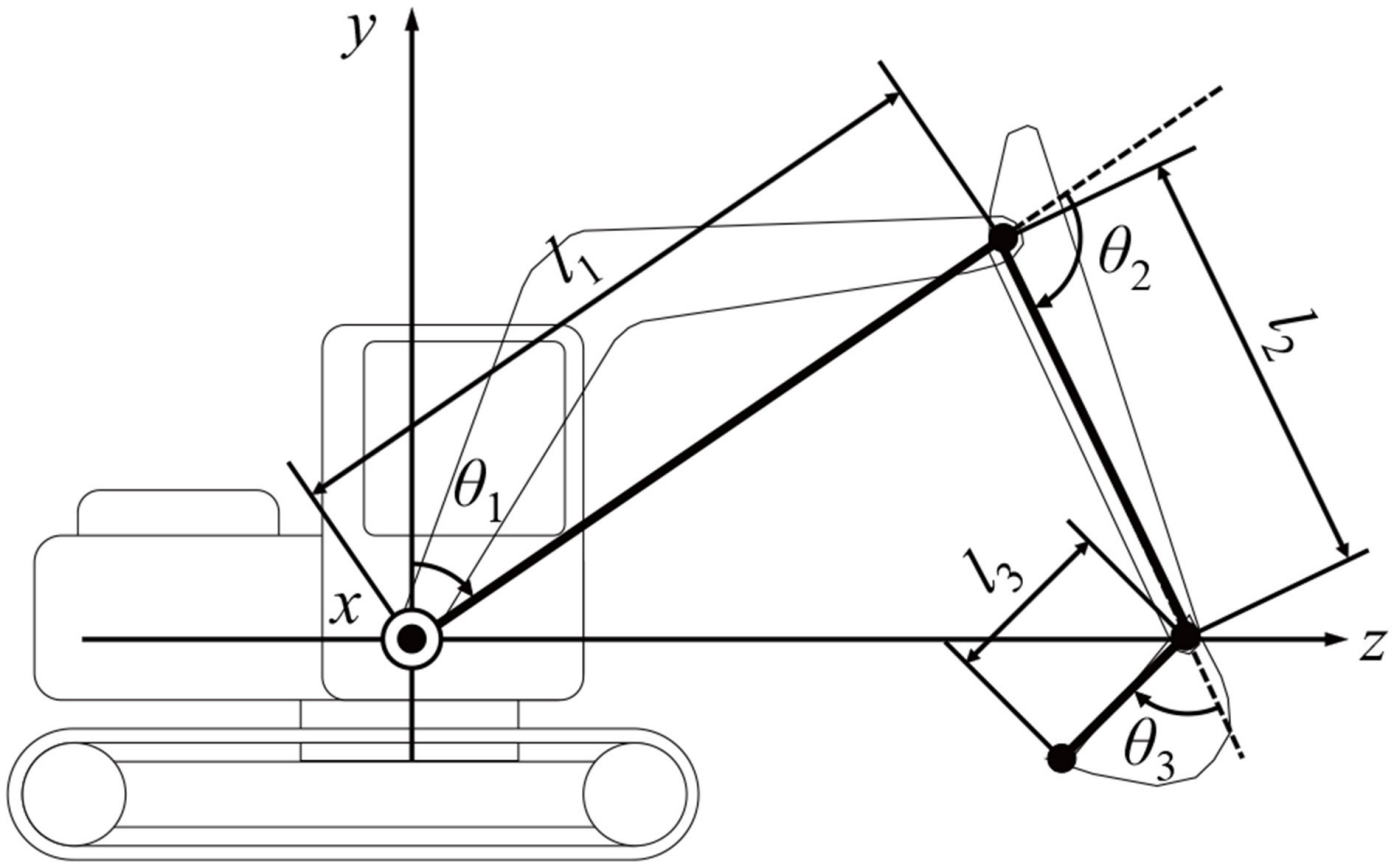
Excavator geometry. *l*_1_, *l*_2_, and *l*_3_ are the lengths of the boom, arm, and bucket, respectively. θ_1_, θ_2_, and θ_3_ are the angles of the boom, arm, and bucket, respectively.

**Figure 6:**
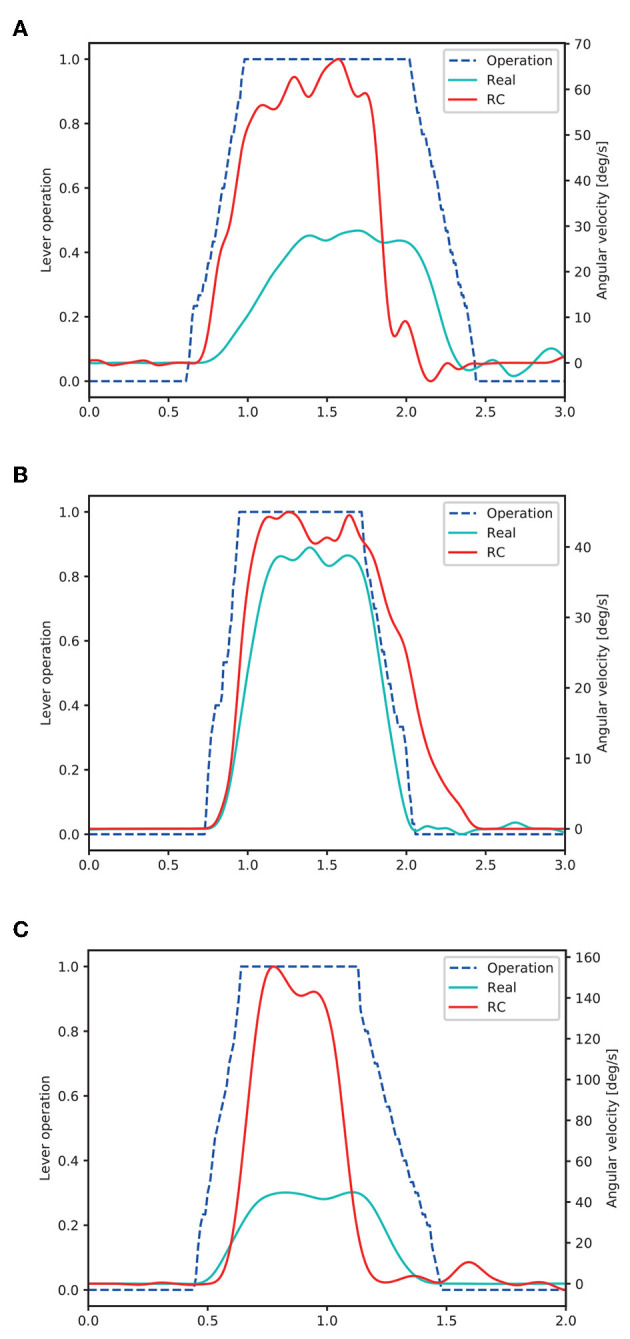
Joint angular velocities of the real excavator and the RC excavator. **(A)** Boom operation, **(B)** arm operation, and **(C)** bucket operation.

To confirm the dynamics of the RC excavator used in the proposed system, we measured the time response of each joint angular velocity of the RC excavator and real excavator (SK135SR-5, Kobelco Construction Machinery) shown in Figure 4. Figure 5 and Table 1 present the initial joint angles. The lever operation performed manually for the real excavator was used as the input to the RC excavator. Figure 6 shows the results of the time response of joint angular velocity. The blue dashed lines represent the lever operation normalized so that the maximum value becomes 1. The cyan and red solid lines represent the joint angular velocity of the real excavator and the RC excavator, respectively.

**Table 1:** Initial joint angles of the excavator (°).

**Operating target**	**θ_1_**	**θ_2_**	**θ_3_**
Boom	45	45	0
Arm	30	60	0
Bucket	30	45	45

We assumed the system as a first-order system with a dead time and estimated the system parameters. The gain *K* was the final value of the angular velocity, and the dead time *L* and the time constant *T* were calculated by the following formulas.

(1) $$\begin{array}{r} L=t_{p}-\frac{v_{p}}{R}. \end{array}$$

(2) $$\begin{array}{r} T=t_{q}-L. \end{array}$$

Here, (*t*_*p*_; *v*_*p*_) is the inflection point of the joint angular velocity during the acceleration. *R* is the slope at the inflection point. Further, *t*_*q*_ is the time when the angular velocity reaches 63.2% of the final value, and *t*_*p*_ and *t*_*q*_ are the times based on the time when the lever input starts.

Table 2 lists the calculated system parameters of each link of the real excavator and the RC excavator, respectively.

**Table 2:** System parameters of each link of excavator.

**Type**	**Operating target**	***K***	***T***	***L***
Real excavator	Boom	29	0.31	0.22
	Arm	39	0.16	0.12
	Bucket	43	0.12	0.07
RC excavator	Boom	63	0.21	0.11
	Arm	43	0.10	0.12
	Bucket	143	0.09	0.15

## 4. Subjective Evaluation of the Operability and Presence of an RC Excavator

In order to investigate the operability of this system and the presence of the images, we employed subjective evaluation questionnaires.

### 4.1. Experiment Protocol

The subjects were eight individuals who had operated an excavator and 12 individuals who had never operated one; informed consent was obtained from the subjects before the experiment. The subjects performed repeated continuous excavation for 3 min. Continuous excavation consisted of four steps: excavating, turning, dumping, and returning. Moreover, continuous excavation began with the extension of the arm of the excavator, and the excavation was performed by digging into the ground. Turning was performed by turning the excavator by 90° while raising its bucket. Dumping was performed by discharging the soil into the bucket. Returning was defined as the operation of returning to the initial state of the excavation. After completing the task, we conducted a subjective evaluation of the operability of this system and the presence of the images by using a questionnaire. The operability was evaluated using six scales of the Japanese version of NASA-TLX (Haga and Mizukami, 1996), which is a subjective evaluation method for a mental workload. The overall workload was calculated using Raw TLX, which uses the average value of all scales. The presence of the images was evaluated using four scales by referring to an evaluation questionnaire for the presence of a wide-field still image (Emoto et al., 2006). The questionnaire items are shown below.

- Operability
  – How much mental and perceptual activity was required?
  – How much physical activity was required?
  – How much time pressure did you feel due to the pace at which the tasks or task elements occurred?
  – How successful were you in performing the task?
  – How hard did you have to work to accomplish your level of performance?
  – How irritated, stressed, and annoyed versus content, relaxed, and complacent did you feel during the task?

- Presence of the images
  – How much do you feel the presence?
  – How much did you feel the powerfulness?
  – How much did you feel the comfort?
  – How much did you feel the depth?

Each item was answered with 0–100 points.

### 4.2. Results

**Figure 7:**
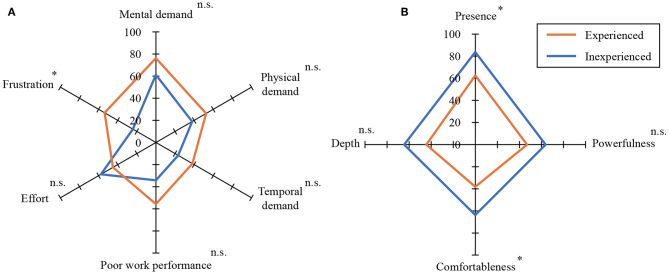
Assessment results for subjective burden and image perception (^*^p < 0.05, n.s., not significant). **(A)** Subjective burden, **(B)** subjective image perception.

**Figure 8:**
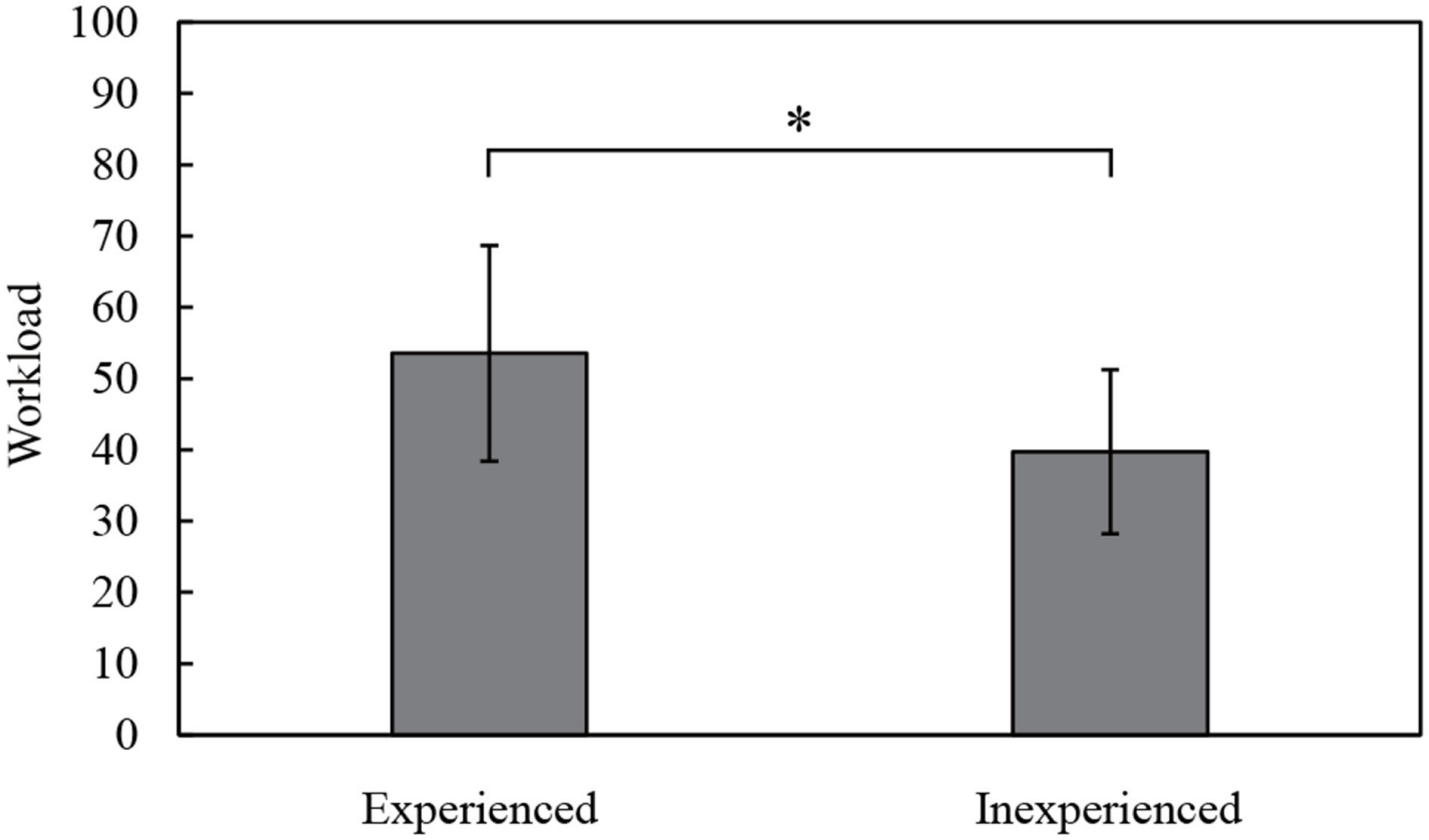
Results of overall workload (Raw TLX, ^*^p < 0.05).

Figure 7A shows the questionnaire results of the subjective evaluation of operability. This radar chart means that the larger the area, the greater is the operational burden. As seen from the graph, the experienced subjects tended to give higher scores than the inexperienced ones for the items other than “effort.” The t-tests between the experienced and inexperienced subjects for each item shows that there is no significant difference in “mental demand,” “physical demand,” “temporal demand,” “poor work performance,” and “effort,” but a significant difference was confirmed for the item “frustration.” Figure 8 shows the overall workload by Raw TLX. This result confirms that the experienced subjects find operating the RC excavator more difficult. Figure 7B shows the questionnaire result of the subjective evaluation of the presence of the images. This radar chart shows that the larger the area, the better is the presence of the images. The graph shows that the experienced subjects tend to give lower scores than the inexperienced subjects for all items. The *t*-tests between the experienced and inexperienced subjects for each item show that there is no significant difference in terms of “powerfulness” and “depth,” but there is a significant difference in the case of “presence” and “comfortableness.” This result confirms that the experienced subjects tend to give lower scores for presence and comfort.

## 5. Evaluation of Excavation Performed by the RC Excavator and Real Excavator

Excavation carried out using the RC excavator and real excavator were evaluated to verify the indices that can be used to quantitatively evaluate the skill of operating a hydraulic excavator, regardless of the operator.

### 5.1. Experiment Protocol

The RC excavator and real excavator (SK135SR-5, Kobelco Construction Machinery) considered in this study are shown in Figures 1, 4, respectively. The subjects were three expert and five non-expert operators. Informed consent was obtained from them before the experiment. In this study, an operator who typically evaluates the operability of a hydraulic excavator was considered as an expert. The subjects performed the following task five times after sufficient practice using the RC excavator and real excavator. The measurement task consisted of three continuous excavation cycles. The second and third cycles were analyzed, and the first cycle starting from the stopped state was excluded. The operation time, operation amount of each lever axis, and each joint angle of the excavator were measured. The angle of the boom, arm, and bucket of the real excavator were calculated from the measured length of the hydraulic cylinder of the excavator, and the swing angle was measured using an angular acceleration sensor. The pilot pressure of each lever of the actual excavator was used as the operation amount of each lever axis. For the RC excavator, the sampling rate of the measurement data was 100 Hz. For the real excavator, the sampling rate of the measurement data was 1,000 Hz, and this was reduced to 100 Hz. The 3D trajectory of the bucket tip was calculated from the joint angle of the excavator by calculating the forward kinematics as follows:

(3) $$\begin{array}{r} \left[\begin{array}{c} x\\ y\\ z \end{array}\right]=\left[\begin{array}{c} \left(l_{1}s_{1}+l_{2}s_{12}+l_{3}s_{123}\right)s_{4}\\ l_{1}c_{1}+l_{2}c_{12}+l_{3}c_{123}\\ \left(l_{1}s_{1}+l_{2}s_{12}+l_{3}s_{123}\right)c_{4} \end{array}\right]. \end{array}$$

(4) $$\{\begin{array}{ll} s_{i} & =\text{sin}\theta_{i},\;\;i=1,4\\ c_{i} & =\text{cos}\theta_{i},\;\;i=1,4\\ s_{12} & =\text{sin}(\theta_{1}+\theta_{2})\\ c_{12} & =\text{cos}(\theta_{1}+\theta_{2})\\ s_{123} & =\text{sin}(\theta_{1}+\theta_{2}+\theta_{3})\\ c_{123} & =\text{cos}(\theta_{1}+\theta_{2}+\theta_{3}) \end{array}.$$

Here, the rightward, upward, and forward directions of the excavator were set as the positive directions of the x, y, and z axes, respectively, as shown in Figure 5. Here, *l*_1_ is the length of the line segment connecting the rotation axis of the boom and the arm's rotation axis; *l*_2_ is the length of the line segment connecting the rotation axis of the arm to the rotation axis of the bucket; *l*_3_ is the length of the line segment connecting the rotation axis of the bucket to the bucket tip; θ_1_ is the angle between the line segment *l*_1_ and the vertical direction; θ_2_ is the angle between the line segment *l*_1_ and segment *l*_2_; θ_3_ is the angle between the line segment *l*_2_ and line segment *l*_3_; and θ_4_ is the angle of the upper revolving body with respect to the lower traveling body. The parameters of the real excavator and the RC excavator as listed in Table 3 were used to calculate the trajectory.

**Table 3:** Length of each link (mm) and movable range of each joint (°) of excavator.

**Type**	***l*_1_**	***l*_2_**	***l*_3_**	***θ*_1_**	***θ*_2_**	***θ*_3_**
Real excavator	5,650	2,940	1,440	28–137	30–159	−41–142
RC excavator	255	125	90	30–100	44–115	0–90

**Figure 9:**
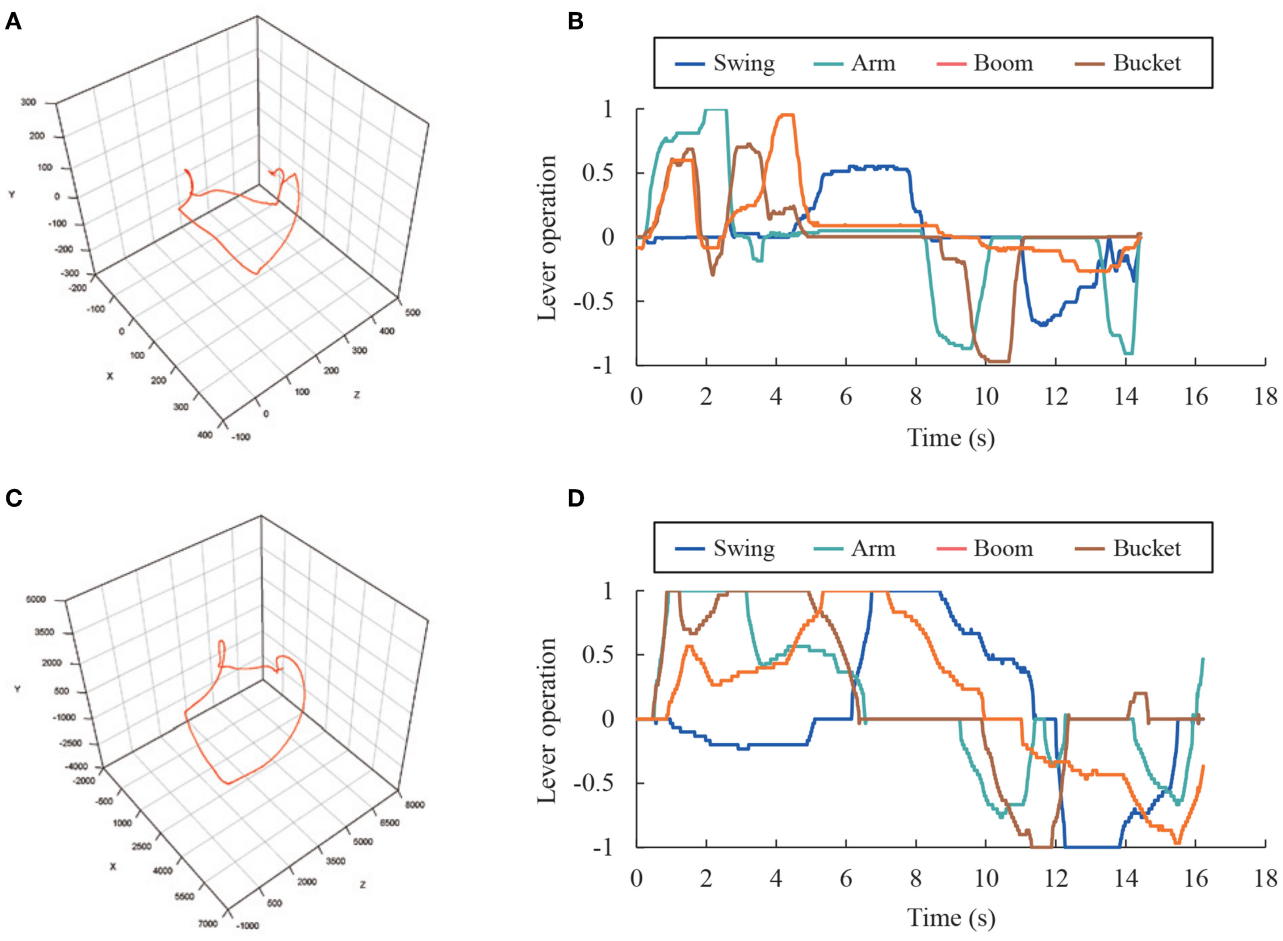
Measurement data during excavation (Expert 1, Task 3, Cycle 1). **(A)** Trajectory of RC excavator bucket (mm). **(B)** Lever operation of RC excavator. **(C)** Trajectory of real excavator bucket (mm). **(D)** Lever operation of real excavator.

Figure 9 shows examples of the measurement trajectory, and the lever operation during one excavation cycle with the RC excavator and real excavator. Here, the lever operation is represented by the time series of the value obtained by normalizing the inclination degree of the turning, boom, arm, and bucket operation levers, from −1 to 1. It was assumed that the excavators differed in terms of the trajectory shape and waveform of the lever operation amount owing to the difference in the ratio of the length of the links and the behavior of the excavator's behavior. These data were calculated using the operation skill evaluation indices.

### 5.2. Calculation of Operation Skill Evaluation Indices

The evaluation indices were the operation time *t*, dispersion of bucket trajectories *d*_*t*_, length of bucket trajectory *l*, average bucket velocity $\bar{v}$, and dispersion of lever operations *d*_*o*_. Each index was calculated for one cycle of the continuous excavation, excavating section, turning section, dumping section, and returning section.

The operation time was calculated because it is typically used as an evaluation index. The dispersion of the bucket trajectory and the dispersion of the lever operation were calculated based on the fact that experts excavate with an approximately unique trajectory, regardless of the excavation order (Sakaida et al., 2008). The trajectory length of the bucket was calculated by considering that the experts had shorter trajectories with more compact operation. The average bucket velocity was calculated by considering that experts performed faster operations. Each index was calculated using the methods described below.

**Figure 10:**
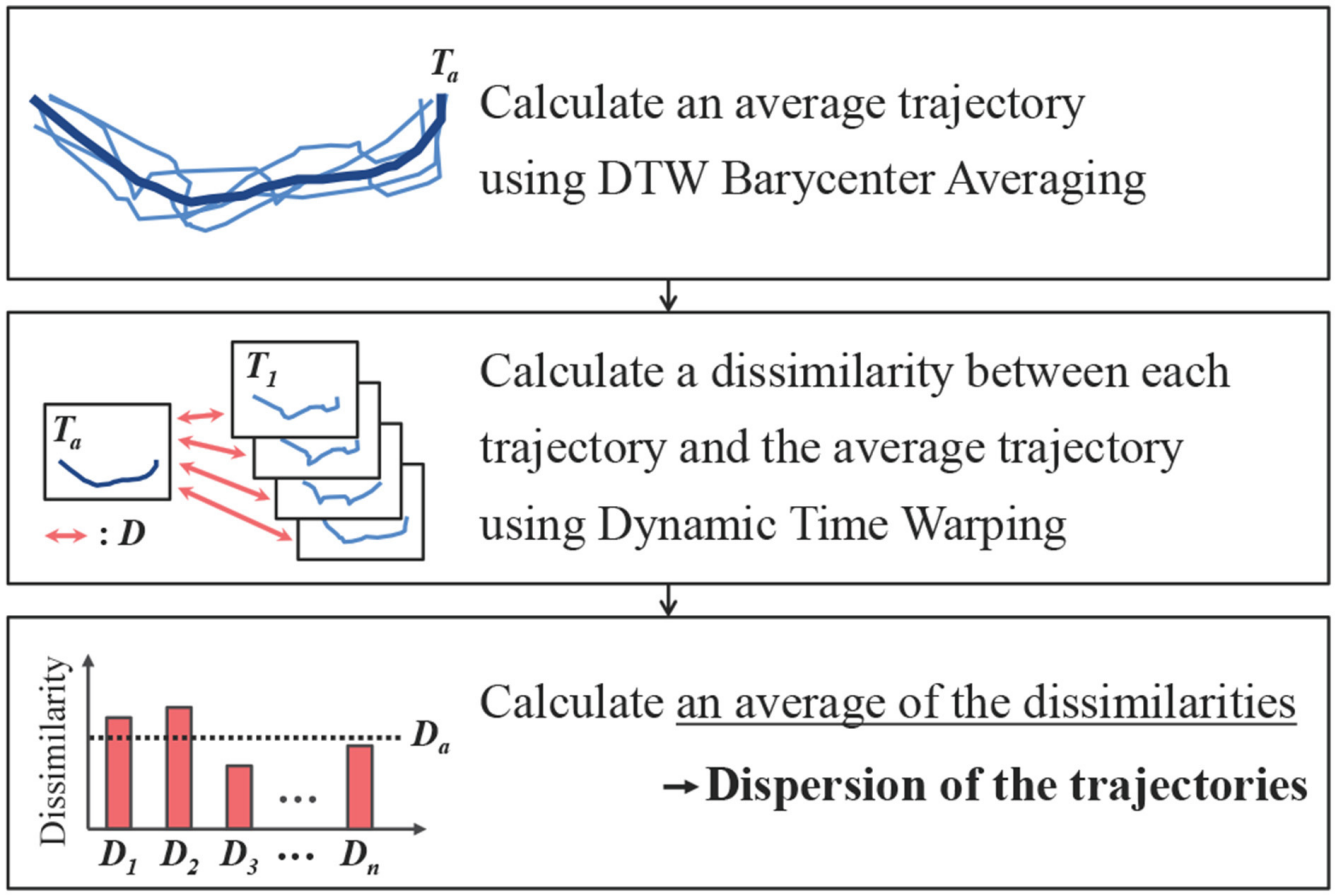
Method of calculating dispersion trajectories.

The dispersion of the bucket trajectories represents the degree of difference among the trajectories, when the same operation was performed multiple times. Figure 10 shows the calculated dispersion flow for the bucket trajectories. First, the average of these trajectories was calculated. However, it was impossible to calculate the average trajectory because the data length was different for each trajectory. Therefore, the average of the trajectories with different data lengths was calculated using the DTW barycenter averaging (DBA) method (Petitjean et al., 2011). Then, the dissimilarity between each trajectory and the average trajectory was calculated. For this purpose, the dynamic time warping (DTW) method (Sakoe and Chiba, 1978), which can calculate the dissimilarity between two time series with different lengths, was adopted. The DTW and DBA are described in the Appendix section. Finally, the average dissimilarity was calculated, and this was considered as the dispersion of the trajectories *d*.

The bucket trajectory length *l* was calculated using the following equation:

(5) $$\begin{array}{r} l=\overset{n-1}{\underset{i=1}{\sum }}\sqrt{{\left(x_{i+1}-x_{i}\right)}^{2}+{\left(y_{i+1}-y_{i}\right)}^{2}+{\left(z_{i+1}-z_{i}\right)}^{2}}. \end{array}$$

Here, (*x*_*i*_, *y*_*i*_, *z*_*i*_) is the *i*-th coordinate of the bucket trajectory with *n* elements.

The average velocity of the bucket $\bar{v}$ was calculated as follows:

(6) $$\begin{array}{r} \bar{v}=\frac{l}{t}. \end{array}$$

The dispersion of the lever operations *d*_*o*_ was calculated in the same manner as *d*_*t*_ for the swing operation, boom operation, arm operation, and bucket operation. Here, the distance function *d*(*a*_*i*_, *b*_*j*_) of the lever operation is defined as follows:

(7) $$\begin{array}{r} d\left(a_{i},b_{j}\right)=|a_{i}-b_{j}|. \end{array}$$

### 5.3. Results

**Figure 11:**
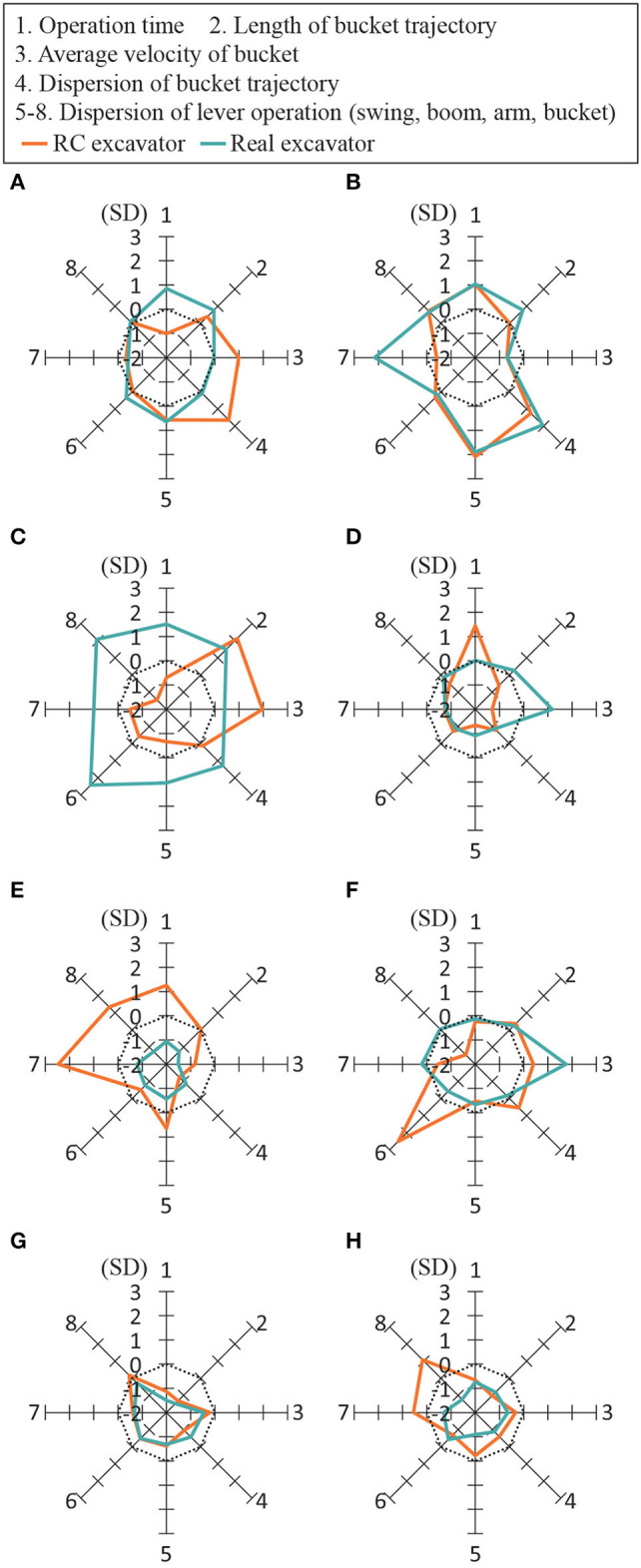
Radar chart of evaluation indices. The standardized reciprocal of each evaluation index was used such that the area of the radar chart became larger for a highly-skilled subject. The dotted line represents the average for all the subjects. **(A**) Expert 1, **(B)** Expert 2, **(C)** Expert 3, **(D)** Non-expert 1, **(E)** Non-expert 2, **(F)** Non-expert 3, **(G)** Non-expert 4, and **(H)** Non-expert 5.

Figure 11 shows the evaluation indices of the RC excavator and the real excavator on the radar chart for each subject. The first axis represents the operation time; the second axis represents the length of the bucket trajectory; the third axis represents the average velocity of the bucket; the fourth axis represents the dispersion of the bucket trajectory; and the fifth to eighth axes represent the dispersion of the lever operation for the swing, boom, arm, and bucket. The standardized reciprocal of each evaluation index was used such that the area of the radar chart was greater for a highly-skilled subject. The dotted line represents the average for all subjects. The figure shows that the average is better than the average in the case that the evaluation index is outside this line. It was confirmed that the experts tended to have a larger radar chart area than the nonexperts for both the RC excavator and the real excavator. The radar chart area for non-expert 3 was larger than that for the other nonexperts, because non-expert 3 had more working experience on operating a hydraulic excavator than the other nonexperts. These results suggest that it is possible to distinguish the skill differences for an expert or non-expert and to distinguish the length of working experience of the hydraulic excavator operator by using the RC excavator. However, the outline of the radar chart of the RC excavator was different from that of the real excavator. This may have been caused by the fact that the behavior of the RC excavator considered in this study differed from that of the real excavator.

## 6. Discussion

The correlation of each evaluation index between the RC excavator and the real excavator was investigated for each section of the continuous excavation. Table 4 lists the calculated result of the correlation between the real excavator and the RC excavator for all evaluation indices. Each row represents an evaluation index, while each column represents the section considered in the calculation. Each value represents the correlation coefficient between the real excavator and RC excavator for each evaluation index. The values with significant correlation are shown in boldface. No significant correlation between the real excavator and the RC excavator data was found in any section of the operation time and dispersion of the swing, arm, and bucket operations. However, in several sections, significant correlation was confirmed for the dispersion of the bucket trajectories, length of the bucket trajectory, average velocity of the bucket, and dispersion of the boom operations. It was thought that the correlation of the dispersion of the bucket trajectories and length of the bucket trajectory is higher because it was unaffected by the difference in the behavior of the excavator. The results suggest that it is possible to partly evaluate the operation characteristics of a real excavator by using an RC excavator whose dynamics differed from those of a real excavator.

**Table 4:** Correlation between real excavator index and RC excavator index (^*^p < 0.05, ^*^^*^p < 0.01).

**Index**	**One cycle**	**Excavating**	**Turning**	**Dumping**	**Returning**
*t*	0.09	−0.10	0.08	−0.26	0.56
*d*_*t*_	^*^0.80	−0.04	^*^0.82	^*^^*^0.87	0.31
*l*	^*^0.73	0.06	^*^0.80	0.35	^*^0.78
$\bar{v}$	0.23	^*^0.76	0.15	0.68	−0.36
*d*_*o*_ (swing)	0.51	−0.38	0.26	0.32	0.60
*d*_*o*_ (boom)	0.35	0.40	^*^0.80	−0.01	−0.29
*d*_*o*_ (arm)	−0.33	−0.25	0.46	0.37	0.02
*d*_*o*_ (bucket)	−0.64	−0.60	0.02	0.63	−0.49

*t is the operation time. d_t_ is the dispersion of bucket trajectories. l is the length of bucket trajectory. $\bar{v}$ is the average bucket velocity. d_o_ is the dispersion of lever operations. The values with significant correlation are shown in boldface*.

Figures 7A, 8 indicate that the experienced operators find it more difficult to operate the RC excavator. Figure 7B indicates that the experienced operators tend to give lower scores for presence and comfort. The reason for this may be that the experienced operators felt a sense of incongruity in the RC excavator that differs in operability and visibility from real excavators. Certainly, the operation interface was reproduced with the gaming joysticks that have different stiffness from the real lever, and the RC excavator was driven by the electric motors instead of hydraulic actuators. Therefore, the operability of the RC excavator was not exactly the same as that of the real excavator. Moreover, we replaced the original human vision by the HMD and omnidirectional camera with much lower resolution than the human eye. The current system was not capable of providing depth information. Nevertheless, the evaluation of depth perception was not much worse because the depth was perceived in a monocular image with the help of environmental conditions such as lighting, shadows, etc. In addition, the latency of the visual system has a great influence on visual immersion. In order to enable more accurate skill evaluation and effective training, it is necessary to closely reproduce the operability and visibility of real excavators in the RC excavator.

The importance of sense of embodiment (SoE) and embodiment of cognition (EoC) in a virtual reality environment (Winn, 2003; Kilteni et al., 2012) was emphasized. Improving SoE and EoC will extend the completeness of the skill evaluation. Multimodal information such as vision, hearing, and haptic sensations can be also used for gaining a better understanding of more advanced skills when operating real excavators. Multimodal information is very important for enhancing EoC and SoE. As future work, we plan to improve the proposed system to display sounds and vibrations during operations.

## 7. Conclusion and Future Work

In this study, we developed a simulator that can measure each joint angle in real time by using an RC excavator with the same viewpoint and operating interface as a real excavator. We calculated evaluation indices for lever operation and bucket movement during excavation using the proposed system and a real excavator. Although the dynamics were largely different, the results showed a high correlation between the RC excavator and real excavator in some operations and suggested that the proposed system could evaluate the operational skills with regard to the correspondence between the direction of the lever and the joint angle of the excavator.

However, not all the operation skills of the real excavator can be learned by training with the RC excavator system developed in this paper. In the future, in order to enable more accurate skill evaluation and effective training, we will attempt to closely reproduce the operability and visibility of a real excavator in the RC excavator. Specifically, we will replace the joysticks with the same levers as those of the real excavator and improve the visual system using an industrial camera with high resolution and low delay. In addition, more evaluation indices are required to evaluate higher-skilled operators. Future work will also include the measurement of the weight of excavated soil and the calculation of the simulated fuel consumption from the measured electric current. This study is limited to the evaluation of the operation skills of hydraulic excavators using the RC excavator, and it has not been verified whether effective training can actually be performed using it. Therefore, we plan to verify the training effect of the RC excavator by observing the changes in the operation skills over time with such training.

## Data Availability Statement

The datasets generated for this study are available on request to the corresponding author.

## Ethics Statement

Written informed consent was obtained from the individual(s) for the publication of any potentially identifiable images or data included in this article.

## Author Contributions

RS designed the study and drafted the manuscript. MI contributed to data collection and interpretation and critically reviewed the manuscript. YK contributed concepts of this research and critically reviewed the manuscript. All authors have approved the final version of the manuscript. Further, all authors agree to be accountable for all aspects of the work in ensuring that questions related to the accuracy or integrity of any part of the work are appropriately investigated and resolved.

### Conflict of Interest

MI, SS, and YY were employed by Kobelco Construction Machinery Co., Ltd. The remaining authors declare that the research was conducted in the absence of any commercial or financial relationships that could be construed as a potential conflict of interest.

## A. Appendix

### A.1. DTW

The DTW is a method of calculating the distance between two time series of different lengths. An example of using the DTW algorithm to calculate the distance of two time series data (*A* = {*a*_1_, *a*_2_, ⋯ , *a*_*I*_}, and *B* = {*b*_1_, *b*_2_, ⋯ , *b*_*J*_}) is presented below.

*Step 1*: The distance *d*(*a*_1_, *b*_1_) between *a*_1_ and *b*_1_ is calculated; this is the DTW distance *D*(1, 1).

*Step 2*: The DTW distance *D*(*i*, 1) is calculated in order as follows:

(A1) $$\begin{array}{r} D\left(i,1\right)=D\left(i-1,1\right)+d\left(a_{i},b_{1}\right). \end{array}$$

(A2) $$\begin{array}{r} i=2,3,\cdots \;,I. \end{array}$$

*Step 3*: The DTW distance *D*(1, *j*) is calculated in order as follows:

(A3) $$\begin{array}{r} D\left(1,j\right)=D\left(1,j-1\right)+d\left(a_{1},b_{j}\right). \end{array}$$

(A4) $$\begin{array}{r} j=2,3,\cdots \;,J. \end{array}$$

*Step 4*: The DTW distance *D*(*i, j*) is calculated in order as follows:

(A5) $$\begin{array}{r} D\left(i,j\right)=min\left\{\begin{array}{c} D\left(i,j-1\right)\\ D\left(i-1,j\right)\\ D\left(i-1,j-1\right) \end{array}\right\}+d\left(a_{i},b_{j}\right). \end{array}$$

(A6) $$\begin{array}{r} i=2,3,\cdots \;,I,\;j=2,3,\cdots \;,J. \end{array}$$

Then, *D*(*I, J*) is the DTW distance of time series *A* and *B*. The smaller this value, the more similar will the two time series data be. The correspondence between the points of time series *A* and *B* was obtained by calculating *D*(*I, J*) and is called the warping path.

In this study, the distance function *d*(*a*_*i*_, *b*_*j*_) could be varied according to the type of time series data. The distance in 3D Euclidean space, which is expressed by the following equation, was used to handle the 3D trajectory data.

(A7) $$\begin{array}{l} d\left(a_{i},b_{j}\right)=\\ \sqrt{{\left(a_{i1}-b_{j1}\right)}^{2}+{\left(a_{i2}-b_{j2}\right)}^{2}+{\left(a_{i3}-b_{j3}\right)}^{2}}. \end{array}$$

(A8) $$\begin{array}{r} a_{i}=\left(a_{i1},a_{i2},a_{i3}\right),\;b_{j}=\left(b_{j1},b_{j2},b_{j3}\right). \end{array}$$

The value obtained by dividing the calculated DTW distance by the number of elements of the warping path was considered as the dissimilarity between the two trajectories to eliminate the influence of the number of elements in the trajectory data.

### A.2. DBA

The DBA is a method of calculating the average time series of multiple time series data. An example of using the algorithm to calculate the average of the three time series (*A* = {*a*_1_, *a*_2_, ⋯ , *a*_*I*_}, *B* = {*b*_1_, *b*_2_, ⋯ , *b*_*J*_}, and *C* = {*c*_1_, *c*_2_, ⋯ , *c*_*K*_}) is presented below.

*Step 1*: Let us consider the arbitrary time series data as the provisional average time series data *M* = {*m*_1_, *m*_2_, ⋯ , *m*_*H*_}.

*Step 2*: The warping path of the average time series data and each time series data are calculated using the DTW method.

*Step 3*: Each point in the average time series is updated to the average value of points in each time series correlated by the warping path.

For example, *m*_*h*_ is updated with the warping path using the following expression with respect to data points *a*_*i*_, *b*_*j*_, and *c*_*k*_ associated with data point *m*_*h*_.

(A9) $$\begin{array}{r} m_{h}=\frac{a_{i}+b_{j}+c_{k}}{3}. \end{array}$$

*Step 4*: Steps 2 and 3 are repeated until the average time series converges.
